# Mitochondrial tRNA modifications: functions, diseases caused by their loss, and treatment strategies

**DOI:** 10.1261/rna.080257.124

**Published:** 2025-03

**Authors:** Takeshi Chujo, Kazuhito Tomizawa

**Affiliations:** Department of Molecular Physiology, Faculty of Life Sciences, Kumamoto University, Kumamoto 860-8556, Japan

**Keywords:** tRNA modification, mitochondrial disease, tRNA modopathy, MELAS, mitoTALEN

## Abstract

Mitochondrial tRNA (mt-tRNA) modifications play pivotal roles in decoding and sustaining tRNA stability, thereby enabling the synthesis of essential respiratory complex proteins in mitochondria. Consequently, loss of human mt-tRNA modifications caused by mutations in the mitochondrial or nuclear genome can cause life-threatening mitochondrial diseases such as encephalopathy and cardiomyopathy. In this article, we first provide a comprehensive overview of the functions of mt-tRNA modifications, the responsible modification enzymes, and the diseases caused by the loss of mt-tRNA modifications. We then discuss progress and potential strategies to treat these diseases, including taurine supplementation for MELAS patients, targeted deletion of mtDNA variants, and overexpression of modification-related proteins. Finally, we discuss factors that need to be overcome to cure “mitochondrial tRNA modopathies.”

## MITOCHONDRIAL tRNA MODIFICATIONS AND THEIR MODIFYING ENZYMES

Mitochondria are eukaryotic organelles that generate the majority of cellular ATP through oxidative phosphorylation (OXPHOS). Human mitochondria contain circular DNAs, called mitochondrial DNAs (mtDNAs), of 16.6 kbp. Human mtDNA is inherited maternally, and is present at ∼100–10,000 copies per cell depending on the cell type and tissue type (Wachsmuth et al. 2016). mtDNA encodes 37 genes: 13 related to respiratory complex proteins, 22 related to mitochondrial tRNAs (mt-tRNAs), and two related to mitochondrial ribosomal RNAs (mt-rRNAs) (Anderson et al. 1981). The 22 mt-tRNAs and two mt-rRNAs are used to synthesize the 13 essential respiratory complex subunits, which assemble together with subunits derived from nuclear DNA to form respiratory complexes I, III, IV, and V. All of the RNA components of the mitochondrial translational apparatus are encoded by mtDNA, whereas all of the protein components are encoded by genes within the nucleus prior to translation in the cytoplasm and importation into the mitochondria (Hallberg and Larsson 2014).

**FIGURE 1.**
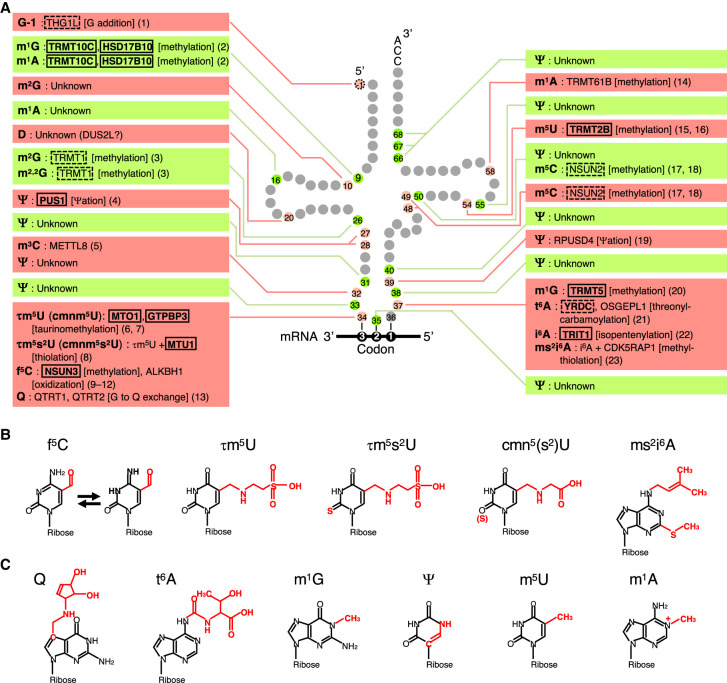
Human mt-tRNA modifications and the responsible modifying enzymes. (*A*) mt-tRNA modifications and the enzymes responsible for these modifications. The name of the modification enzyme (or its partner protein) and the type of reaction are presented alongside the species of tRNA modification. Proteins harboring pathogenic mutations are enclosed within rectangles. Proteins that harbor pathogenic mutations, but the disease caused is likely due to loss of cytoplasmic tRNA modifications, are enclosed within dotted rectangles. Abbreviations for RNA modifications: G–1, posttranscriptional G addition at 5′; m^1^G, *N*^*1*^-methylguanosine; m^1^A, *N*^*1*^-methyladenosine; m^2^G, *N*^*2*^-methylguanosine; m^2,2^G, *N*^*2*^,*N*^*2*^-dimethylguanosine; Ψ, pseudouridine; m^3^C, 3-methylcytidine; τm^5^U, 5-taurinomethyluridine; τm^5^s^2^U, 5-taurinomethyl-2-thiouridine; cmnm^5^U, 5-carboxymethylaminomethyluridine; cmnm^5^s^2^U, 5-carboxymethylaminomethyl-2-thiouridine; f^5^C, 5-formylcytidine; Q, queuosine; m^5^U, 5-methyluridine; m^5^C, 5-methylcytidine; t^6^A, *N*^*6*^-threonylcarbamoyladenosine; i^6^A, *N*^*6*^-isopentenyladenosine; ms^2^i^6^A, 2-methylthio-*N*^*6*^-isopentenyladenosine. References: 1 (Nakamura et al. 2018), 2 (Vilardo et al. 2012), 3 (Dewe et al. 2017), 4 (Patton et al. 2005), 5 (Scholler et al. 2021), 6 (Fakruddin et al. 2018), 7 (Asano et al. 2018), 8 (Umeda et al. 2005), 9 (Nakano et al. 2016), 10 (Van Haute et al. 2016). 11 (Kawarada et al. 2017),12 (Haag et al. 2016),13 (Suzuki et al. 2020),14 (Chujo and Suzuki 2012), 15 (Powell and Minczuk 2020), 16 (Laptev et al. 2020), 17 (Shinoda et al. 2019), 18 (Van Haute et al. 2019), 19 (Zaganelli et al. 2017), 20 (Brule et al. 2004), 21 (Lin et al. 2018), 22 (Yarham et al. 2014), 23 (Wei et al. 2015). (*B*) Mammalian mt-tRNA modifications that are present only in mt-tRNAs and not in cytoplasmic tRNAs. (*C*) Other mt-tRNA modifications relevant to either the onset or potential treatment of the mitochondrial diseases referred to in this manuscript.

The 22 human mt-tRNAs contain 18 kinds of chemically modified nucleotide at 137 positions; these modified nucleotides make up 8.7% of the nucleotides within mt-tRNAs (Fig. 1A–C; Suzuki et al. 2020). Of these mt-tRNA modifications, six are present only in mt-tRNAs and not in cytoplasmic tRNAs (Fig. 1B). All tRNA modifications are introduced posttranscriptionally at specific tRNA positions by specific mt-tRNA modification enzymes. To date, 22 mt-tRNA modification enzymes or their partner proteins have been identified (Fig. 1A), and approximately 10 additional modification enzymes likely exist. Pathogenic mutations have been identified in 14 genes encoding mt-tRNA modification enzymes (Fig. 1A). These mutations cause mitochondrial diseases, which are characterized by a genetic defect that predominantly affects mitochondrial OXPHOS (Gorman et al. 2016). Mitochondrial diseases can be mild or severe, and mutations in mt-tRNA modification enzymes that generate anticodon modifications often cause the most severe symptoms (Table 1). Moreover, mitochondrial diseases are often caused by mutations in mtDNA, particularly in mt-tRNA genes, which can result in the loss of important mt-tRNA anticodon modifications (Table 1; Tomizawa and Wei 2020). Therefore, to better understand the pathogenic mechanisms underlying “mt-tRNA modopathies,” or mitochondrial diseases caused by deficiency of mt-tRNA modifications, we need to ascertain how anticodon modifications support decoding. Thus, the next section will explain the roles of tRNA modifications within the context of decoding.

**TABLE 1.** Mitochondrial diseases caused by loss of mt-tRNA modifications

Mutated gene	Modification (tRNA position)	Disease	Reference
*mt-tRNA* ^*Leu(UUR)*^	τm^5^U (34)	MELAS (mitochondrial myopathy, encephalopathy, lactic acidosis, stroke-like episodes); MIDD (mitochondrial inherited diabetes and deafness)	Goto et al. 1990, 1991; Van den Ouweland et al. 1992; Yasukawa et al. 2000b; Kirino et al. 2005
*mt-tRNA* ^*Lys*^	τm^5^U (34)	MERRF (myoclonic epilepsy with ragged red fibers)	Schoffner et al. 1990; Yasukawa et al. 2000a
*MTO1*	τm^5^U (34)	Hypertrophic cardiomyopathy, lactic acidosis, developmental delay, seizures, optic atrophy, ataxia	Ghezzi et al. 2012; O'Byrne et al. 2018
*GTPBP3*	τm^5^U (34)	Hypertrophic cardiomyopathy, lactic acidosis, encephalopathy	Kopajtich et al. 2014
*MTU1*	(τm^5^)s^2^U (34)	RILF (reversible infantile liver failure)	Zeharia et al. 2009
*NSUN3*	f^5^C (34)	Microcephaly, muscular weakness, developmental delay, encephalopathy, seizures	Van Haute et al. 2016; Paramasivam et al. 2020
*TRMT5*	m^1^G (37)^a^	Cardiomyopathy, muscular weakness, lactic acidosis, neuropathy	Powell et al. 2015
*TRIT1*	i^6^A (37)^a^	Microcephaly, muscular weakness, epilepsy, developmental delay	Yarham et al. 2014; Kernohan et al. 2017
*PUS1*	Ψ (27, 28)^a^	MLASA (myopathy, lactic acidosis, and sideroblastic anemia)	Patton et al. 2005
*TRMT2B*	m^5^U (54)^b^	ALS (amyotrophic lateral sclerosis)	Liu et al. 2024
*TRMT10C*	m^1^G, m^1^A (9)^c^	Lactic acidosis, muscular weakness, feeding difficulties, deafness	Metodiev et al. 2016
*HSD17B10*	m^1^G, m^1^A (9)^c^	Neurodegeneration, cardiomyopathy	Oerum et al. 2017

^a^Also responsible for the same modification in cytoplasmic tRNAs.

^b^Also responsible for m^5^U modification in 12S rRNA.

^c^Components of the mitochondrial RNaseP used for 5′ processing of mt-tRNAs.

## UNIQUE MITOCHONDRIAL GENETIC CODES ARE DECODED USING mt-tRNA ANTICODON MODIFICATIONS IN A MINIMUM SET OF 22 tRNAs

**FIGURE 2.**
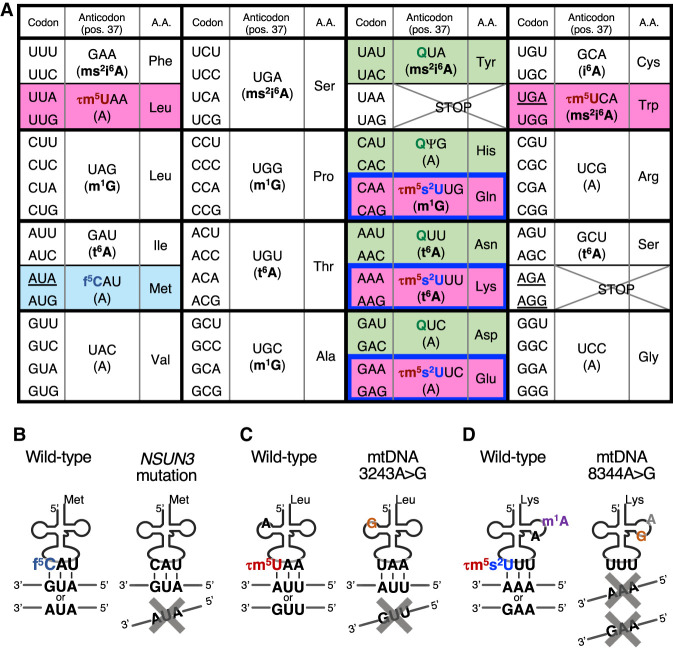
Human mitochondrial codons are decoded using tRNA anticodon modifications. (*A*) Table of human mitochondrial codons. Codons, encoded amino acids, anticodon sequences (including the wobble modification), and the position 37 nt, are shown. Colored two-codon boxes: pink box decoded using τm^5^U-modified tRNAs; light blue box decoded using f^5^C-modified tRNA; green box decoded using Q-modified tRNAs; and blue framed box decoded using (τm^5^)s^2^U-modified tRNAs. Four codons that differ from the canonical genetic codes are underlined. (*B*) Decoding AUR codons as Met enabled by f^5^C modifications in the mt-tRNA^Met^ wobble position. (*C*) Decoding of UUR codons as Leu enabled by the τm^5^U modification in the mt-tRNA^Leu(UUR)^ wobble position. MELAS-causing mtDNA 3243 A > G mutation impedes MTO1–GTPBP3 complex from incorporating the τm^5^U modification. (*D*) Decoding of AAR codons as Lys enabled by the τm^5^s^2^U modification in the mt-tRNA^Lys^ wobble position. MERRF-causing mtDNA 8344 A > G mutation impedes MTO1–GTPBP3 complex and MTU1 from incorporating the τm^5^s^2^U modification and also impedes TRMT61B from incorporating the m^1^A modification.

The mammalian mitochondrial genetic code deviates from the canonical genetic code with respect to four codons: AUA codes for Met instead of Ile; UGA codes for Trp instead of a termination codon; and AGA and AGG code for termination instead of arginine (Fig. 2A, underlined codons). In mammalian mitochondria, the 60 sense codons are decoded by only 22 mt-tRNAs, which is the minimum set of tRNAs required for a decoding system in any organism or organelle (Suzuki et al. 2011). For comparison, the number of isoacceptors (tRNAs with different anticodons) is 46 for human cytoplasmic tRNAs, 42 for yeast cytoplasmic tRNAs, and 41 for *Escherichia coli* tRNAs (Chan and Lowe 2016).

During the decoding process, the codon triplet base pairs with the three anticodon bases of the tRNA at positions 36, 35, and 34 (Fig. 1A). Codon positions 1 and 2 base-pair with tRNA positions 36 and 35 via normal Watson–Crick base-pairing (i.e., A–U and G–C). In contrast, in mitochondria, an unmodified U at tRNA position 34 (the wobble position) allows any of A, U, G, or C in the third nucleotide position of the mRNA codon, thereby enabling tRNAs with an unmodified U at the wobble position to decode four-codon boxes (Fig. 2A; Suzuki et al. 2011) and minimizing the number of isoacceptors of mt-tRNAs to 22. In contrast, to decode two-codon boxes, modified bases at the wobble position and at position 37 play critical roles, which are explained below.

First, to decode both an AUG codon and an AUA codon as methionine, mt-tRNA^Met^ bears a 5-formylcytidine modification (f^5^C, Fig. 1B) in the wobble position (Moriya et al. 1994). f^5^C enables the anticodon f^5^CAU of mt-tRNA^Met^ to base-pair with, and efficiently translate, not only the AUG codon but also the AUA codon in human mitochondria (Fig. 2B; Takemoto et al. 2009; Wakigawa et al. 2024). f^5^C enables f^5^C–A pairing by promoting imino-oxo tautomerization of the cytosine base (Fig. 1B; Cantara et al. 2013); thus, f^5^C modification expands base-pairing capacity. f^5^C is synthesized by two enzymes: NSUN3 and ALKBH1. After mt-tRNA^Met^ is transcribed, NSUN3 first methylates cytidine to form 5-methylcytidine, and ALKBH1 then oxidizes the methyl group to form a formyl group (Haag et al. 2016; Nakano et al. 2016; Van Haute et al. 2016; Kawarada et al. 2017). The physiological importance of f^5^C is demonstrated by the embryonic lethality of *Nsun3* KO mice between E10.5 and E12.5 (Table 2; Murakami et al. 2023). Moreover, mutations in *NSUN3* cause mitochondrial diseases from infants (Table 1; Van Haute et al. 2016; Paramasivam et al. 2020). We note that similar to many other tRNA modification enzymes that are important for body development, NSUN3 also supports the progression of cancer; NSUN3-mediated mitochondrial translation is required for metastasis-initiating tumor cells to activate invasion and dissemination (Delaunay et al. 2022).

**TABLE 2.** mt-tRNA-modifying enzyme knockout mice

Mouse model	Modification (tRNA position)	Phenotype	Reference
*Mto1* Knockout (KO)	τm^5^U (34)	Embryonic lethal before E9, cytoplasmic protein aggregation, and unfolded protein stress in KO cells	Fakruddin et al. 2018
*Mto1* heart KO	τm^5^U (34)	Death within 24 h after birth	Fakruddin et al. 2018
*Mtu1* KO	(τm^5^)s^2^U (34)	Embryonic lethal before E9	Wu et al. 2016
*Mtu1* liver KO	τm^5^s^2^U (34)	Liver injury	Wu et al. 2016
*Nsun3* KO	f^5^C (34)	Embryonic lethal between E10.5 and E12.5	Murakami et al. 2023
*Nsun3* heart KO	f^5^C (34)	Mild heart enlargement	Murakami et al. 2023
*Pus1* KO	Ψ (27, 28)	Reduced exercise capacity, changes in myosin heavy chain ratio	Mangum et al. 2016
*Cdk5rap1* KO	ms^2^i^6^A (37)	Reduced exercise capacity	Wei et al. 2015
*Osgepl1* KO	t^6^A (37)	No detectable tissue phenotype	Zhang et al. 2024
*Qtrt1* KO	Q (34)	No reported mitochondria-related phenotype	Cirzi et al. 2023
*Nsun2* KO	m^5^C (48, 49, 50)	No reported mitochondria-related phenotype	Blanco et al. 2014; Shinoda et al. 2019

KO mice without the tissue name being mentioned are whole-body KO mice.

Second, five mt-tRNAs that decipher five NNR two-codon boxes (N = A, U, G, C; R = purine = A or G) contain 5-taurinomethyluridine (τm^5^U), or its 2-thio derivative τm^5^s^2^U (Fig. 1B), at their wobble positions (Suzuki et al. 2002). These five mt-tRNAs, i.e., mt-tRNA^Leu(UUR)^, mt-tRNA^Gln^, mt-tRNA^Lys^, mt-tRNA^Glu^, and mt-tRNA^Trp^, decode all of the NNR two-codon boxes except the Met-encoding AUR codon box (the pink boxes in Fig. 2A). In addition, mt-tRNA^Gln^, mt-tRNA^Lys^, and mt-tRNA^Glu^ are further 2-thiolated to yield τm^5^s^2^U. Generally, when xm^5^s^2^U (a derivative of 5-methyl-2-thiouridine) is at the wobble position, it largely fixes the ribose in the C3′-endo form, leading to preferential base-pairing with A- or G-ending codons and not to U- or C-ending codons (Yokoyama et al. 1985; Johansson et al. 2008; Suzuki et al. 2011). Moreover, the τm^5^U modification stabilizes τm^5^U–G base-pairing by converting it to a Watson–Crick-like form (Kirino et al. 2005; Kurata et al. 2008). Consequently, loss of τm^5^U from mt-tRNA^Leu(UUR)^, which is caused by a mutation in the *mt-tRNA*^*Leu(UUR)*^ gene, means that UUG codons cannot be translated efficiently (Fig. 2C), resulting in severe mitochondrial diseases and diabetes (Table 1; Goto et al. 1990; Van den Ouweland et al. 1992; Kirino et al. 2005). The τm^5^U modification is incorporated by the MTO1–GTPBP3 complex (Asano et al. 2018; Fakruddin et al. 2018), and the 2-thiolation in τm^5^s^2^U is installed by MTU1 (Umeda et al. 2005). τm^5^U is synthesized by incorporating a taurine moiety and a methylene moiety, derived from taurine and 5,10-methylene-tetrahydrofolate, respectively (Asano et al. 2018). Thus, upon depletion of taurine, τm^5^U and τm^5^s^2^U are lost and, interestingly, glycine is incorporated instead, resulting in 5-carboxymethylaminomethyluridine (cmn^5^U) and its 2-thio derivative cmnm^5^s^2^U (Fig. 1B; Asano et al. 2018). *Mto1* KO mice and *Mtu1* KO mice are embryonic lethal before E9, showing the strongest phenotype of all mt-tRNA modification enzyme KO mice (Table 2; Wu et al. 2016; Fakruddin et al. 2018). Loss of taurine modifications caused by mutations in *MTO1*, *GTPBP3*, *mt-tRNA*^*Leu(UUR)*^, or *mt-tRNA*^*Lys*^ genes causes various mitochondrial diseases, which will be described in a later section.

Third, queuosine (Q, Fig. 1C) is present at the wobble positions of tRNAs, corresponding to four of the NAY (Y = pyrimidine = U or C) two-codon boxes encoding Tyr, His, Asn, and Asp (the green boxes in Fig. 2A). In human cells, Q in mt-tRNA^Tyr^ supports efficient decoding of the UAU codon (Suzuki et al. 2020). Q is installed by base exchange of wobble G with queuine, mediated by a tRNA guanine transglycosylase comprising QTRT1 and QTRT2 proteins (Suzuki et al. 2020). Unlike mice in which other wobble modification enzymes are knocked out, *Qtrt1* KO mice show no mitochondria-related phenotype (Cirzi et al. 2023), a finding consistent with the absence of reports about diseases caused by loss-of-function mutations in human *QTRT1* or *QTRT2*.

In mammalian mitochondria, tRNA position 37, located at the 3′ side of the anticodon, bears modifications such as *N*^*6*^-threonylcarbamoyladenosine (t^6^A), *N*^*6*^-isopentenylcarbamoyladenosine (i^6^A), the 2-methylthio derivative of i^6^A (ms^2^i^6^A), and *N*^*1*^-methylguanosine (m^1^G) (Figs. 1B,C and 2A). Generally, modifications at position 37 stabilize codon–anticodon pairing and maintain the reading frame by increasing base-stacking interactions and/or by preventing unwanted base-pairing within the anticodon (Bjork et al. 1989; Wilson and Roe 1989; Dao et al. 1994; Wei et al. 2011). OSGEPL1 and YRDC are responsible for the t^6^A modification in mt-tRNAs (Lin et al. 2018). Whereas *Osgepl1* KO mice show no detectable phenotype, *OSGEPL1* KO cells show reduced mitochondrial translation and slowdown of mitoribosomes at lysine codons (Lin et al. 2018; Wakigawa et al. 2024; Zhang et al. 2024). CDK5RAP1 is responsible for 2-methylthio modification of ms^2^i^6^A, and *Cdk5rap1* KO mice show reduced mitochondrial translation and reduced activity of respiratory complexes I and IV (Wei et al. 2015).

## MITOCHONDRIAL DISEASES CAUSED BY LOSS OF tRNA MODIFICATIONS

Mitochondrial diseases, a group of heterogeneous genetic disorders related to defects in OXPHOS (Gorman et al. 2016), are caused by mutations in nuclear DNA or mtDNA; the prevalence of such diseases is >1 in 5000 births (Haas et al. 2007). The prevalence of adult patients with mitochondrial disease caused by mutations in mtDNA or nuclear DNA is approximately 20 per 100,000 individuals and three per 100,000 individuals, respectively (Gorman et al. 2015). Mitochondrial translation synthesizes the 13 subunits essential for respiratory complexes I, III, IV, and V. Consequently, loss of important mt-tRNA modifications results in decreased OXPHOS, leading to increased glycolysis to sustain ATP levels. This results in the accumulation of lactate and NADH, and a decrease in NAD^+,^ as well as an increase in reactive oxygen species (ROS), which may lead eventually to inflammation and/or cell death (Zhou and Tian 2018; Chujo and Tomizawa 2021). In some cases, loss of mt-tRNA modifications decreases the import of mitochondrial proteins, accompanied by accumulation and aggregation of mitochondrial proteins in the cytoplasm, thereby triggering the unfolded protein response (Fakruddin et al. 2018). In particular, ATP synthesis is required by energy-consuming tissues such as the brain and heart. Thus, loss of important tRNA modifications most frequently manifests as encephalopathy and/or cardiomyopathy, resulting in severe disability and shortened life span (Gorman et al. 2015).

To develop strategies to treat/cure such diseases, we must gain insight into the molecular pathogenesis underlying each individual mt-tRNA modopathy. In the following sections, we will describe the molecular pathogenic mechanisms underlying mitochondrial diseases caused by mutations in genes encoding mt-tRNA and mt-tRNA modification enzymes.

## MELAS AND MIDD

One of the most common pathogenic mutations among all mitochondrial diseases is the 3243A > G mutation in mtDNA (Gorman et al. 2015). Position 3243 in mtDNA corresponds to position 14 in mt-tRNA^Leu(UUR)^, which is located within the D loop; thus, the mutation may disrupt the U8–A14 hydrogen bonding present in the canonical tRNA structure. The mtDNA 3243A > G mutation is observed in ∼80% of individuals with mitochondrial myopathy, encephalopathy, lactic acidosis, and stroke-like episodes (MELAS) (Table 1; Goto et al. 1990). Another 10% of patients with MELAS harbor a 3271T > C mutation, which is also located in the *mt-tRNA*^*Leu(UUR)*^ gene (Goto et al. 1991). The MELAS mutations in *mt-tRNA*^*Leu(UUR)*^ decrease the processing, aminoacylation, and stability of mt-tRNA^Leu(UUR)^ (Yasukawa et al. 2000b; Levinger et al. 2004). Moreover, and importantly, 3243A > G, 3271T > C and other MELAS-associated mutations result in loss of the τm^5^U modification in mt-tRNA^Leu(UUR)^ (Yasukawa et al. 2000b; Kirino et al. 2005). These data strongly suggest that MELAS mutations impede the ability of the MTO1–GTPBP3 complex to modify mt-tRNA^Leu(UUR)^. Without the τm^5^U modification, mt-tRNA^Leu(UUR)^ containing anticodon UAA fails to decode the leucine UUG codon (Fig. 2C; Kirino et al. 2004). As a result, mitoribosomes often slow down at the UUG codon, resulting in translational defects; this is particularly true for *ND6* mRNA (Kirino et al. 2004; Wakigawa et al. 2024), which contains the most UUG codons. Thus, decreased translation of the UUG codon and ND6 (a subunit of complex I) in MELAS cells may explain why reduction of complex I activity is a major biochemical feature of MELAS (Goto et al. 1992).

The mtDNA 3243A > G mutation also causes mitochondrial inherited diabetes and deafness (MIDD) (Table 1; Van den Ouweland et al. 1992). Pancreatic beta cells, which synthesize insulin, are highly dependent on mitochondrial ATP synthesis. The exact pathogenic mechanism underlying MIDD is unclear, but is thought to be in part due to aberrant mitochondrial production of excess ROS, which stresses the pancreatic beta cells and leads eventually to apoptosis (Supale et al. 2012). As for deafness, atrophy of stria vascularis in cochlea was reported as the cause of deafness in a mtDNA 3243A > G patient (Handzel et al. 2020). Additionally, cochlear implants resulted in positive hearing outcomes in mtDNA 3243A > G patients (Crundwell et al. 2022).

## MERRF

Myoclonus epilepsy associated with ragged red fibers (MERRF) is another well-characterized clinical subgroup of mitochondrial encephalopathy (Fukuhara et al. 1980). Many MERRF patients carry a mtDNA 8344A > G mutation in the *mt-tRNA*^*Lys*^ gene (Schoffner et al. 1990). The mutant mt-tRNA^Lys^ harbors somewhat reduced aminoacylation levels, but the major effect is loss of τm^5^s^2^U (Yasukawa et al. 2000a, 2001, 2005). The hypomodification prevents mt-tRNA^Lys^ from efficiently decoding the AAA and AAG codons, leading to a defect in mitochondrial translation (Fig. 2D; Yasukawa et al. 2001). In addition, the MERRF mutation causes loss of 1-methyladenosine (m^1^A, Fig. 1C) at position 58 of mt-tRNA^Lys^, which is incorporated by TRMT61B (Chujo and Suzuki 2012; Richter et al. 2018).

## RILF

**FIGURE 3.**
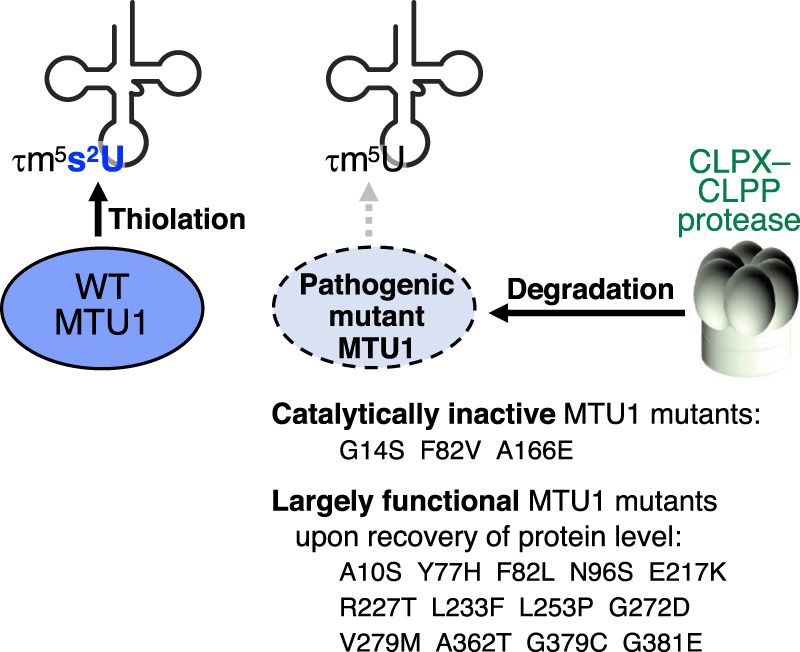
MTU1 protein degradation caused by pathogenic mutations associated with RILF. MTU1 is responsible for 2-thiolation of the τm^5^s^2^U modification. The 16 pathogenic MTU1 mutants that cause RILF are degraded in mitochondria by the CLPX–CLPP protease. Among the 16 pathogenic MTU1 mutants, three are catalytically inactive, and 13 can restore 2-thiolation on mt-tRNAs upon overexpression (Ahmad et al. 2024).

Reversible infantile liver failure (RILF) is an acute and transient type of liver failure that occurs during the first year after birth, occasionally accompanied by cardiomyopathy and other peripheral symptoms. RILF is often caused by mutation of *MTU1* (Zeharia et al. 2009), which encodes a mt-tRNA-specific 2-thiouridylase (Umeda et al. 2005). Most severe cases of RILF result in death; however, if RILF patients survive the acute phase, they recover fully. It is not clear why liver failure is acute and transient. More than 30 missense mutations in MTU1 have been identified (Zeharia et al. 2009; Vogel et al. 2023). A study of 16 pathogenic MTU1 mutants revealed that the steady-state level of various pathogenic MTU1 mutant proteins was decreased markedly due to accelerated degradation by the mitochondrial CLPX–CLPP protease complex (Fig. 3; Ahmad et al. 2024). Whereas three out of the 16 pathogenic MTU1 mutants lost 2-thiolation activity completely, 13 retained at least partial 2-thiolase activity when the protein level was recovered by exogenous expression of mutant *MTU1* or knockdown of *CLPP* (Ahmad et al. 2024). Patient survival was clearly dependent on residual 2-thiolation activity, with a threshold of 10% 2-thiolation needed for survival and recovery from RILF (Ahmad et al. 2024). Additionally, pathogenic MTU1 variants that manifested not only RILF but also cardiomyopathy tended to have lower 2-thiolation activity (Vogel et al. 2023; Ahmad et al. 2024).

## OTHER MITOCHONDRIAL DISEASES CAUSED BY MUTATIONS OF mt-tRNA-MODIFYING ENZYMES

Pathogenic mutations have been identified in 13 proteins responsible for mt-tRNA modifications: TRMT10C, HSD17B10, TRMT1, PUS1, MTO1, GTPBP3, MTU1, NSUN3, TRMT5, YRDC, TRIT1, NSUN2, and TRMT2B (Fig. 1A). Of these, mutations in eight proteins, i.e., PUS1, MTO1, GTPBP3, MTU1, NSUN3, TRMT5, TRIT1, and TRMT2B are considered to cause mitochondrial diseases (based on disease symptoms and/or subcellular protein localization). Symptoms caused by mutations in TRMT1, YRDC, or NSUN2 suggest that the diseases are likely caused by loss of cytoplasmic tRNA modifications, for which these proteins are also responsible. TRMT10C and HSD17B10, which together form a complex that incorporates m^1^G or m^1^A at position nine (Vilardo et al. 2012), are also components of mitochondrial RNaseP, which cleaves the 5′ end of nascent mt-tRNAs (Holzmann et al. 2008); thus, regarding the disease onset, it is difficult to dissect the effect of reduced mt-tRNA modification and the effect of reduced mitochondrial RNA processing.

Pseudouridine synthase 1 (PUS1) is responsible for generating pseudouridines (Ψ, Fig. 1C) at positions 27 and 28. Mutations in *PUS1* cause myopathy, lactic acidosis, and sideroblastic anemia (MLASA) (Patton et al. 2005). Ψ is a C–C glycosidic isomer of uridine that exposes *N*^*1*^ nitrogen, which then bridges with the phosphate backbone via a water molecule. This causes the ribose to adopt a C3′-endo conformation and improves base-stacking in a helical environment. As a result, Ψ in a helix confers a higher melting temperature (Arnez and Steitz 1994; Davis 1995; Yarian et al. 1999; Jones et al. 2006; Sipa et al. 2007). Thus, although not investigated specifically in the context of mt-tRNA, PUS1-mediated insertion of Ψ into the anticodon stem might stabilize the helical structure.

Mutations in tRNA methyltransferase 2B (TRMT2B) have been identified in patients with amyotrophic lateral sclerosis (ALS) (Liu et al. 2024). TRMT2B installs 5-methyluridine (m^5^U, Fig. 1C) at mt-tRNA position 54 (Laptev et al. 2020; Powell and Minczuk 2020). ALS patients have decreased protein levels of NADH dehydrogenase subunit 1 (an essential subunit of complex I), diminished complex I activity, lower mitochondrial respiration, and increased ROS levels (Liu et al. 2024). In addition to m^5^U in mt-tRNAs, TRMT2B also incorporates m^5^U into mt 12S rRNA (Laptev et al. 2020; Powell and Minczuk 2020). Thus, it is unclear how much the loss of mt-tRNA m^5^U and/or mt-rRNA m^5^U is responsible for ALS; this will need to be investigated using methods such as mitoribosome profiling.

## STRATEGIES FOR AND PROGRESS TOWARD CURING mt-tRNA MODOPATHIES

Currently, there is no widely used, fundamental curative treatment for mitochondrial diseases; therefore, clinical management focuses on treating the complications (Russell et al. 2020). There has been much effort directed at curing mt-tRNA modopathies; indeed, one treatment has shown some effects in MELAS patients, and other measures show effects at the cell culture level. Three main strategies are aimed at curing mt-tRNA modopathies: (1) removal of pathogenic mtDNA using a sequence-specific DNase; (2) supplementation with a substrate used by modification enzymes; and (3) exogenous expression of modification enzymes or tRNA protection proteins (Table 3).

**TABLE 3.** Characteristics of current methods having potential for the therapy of mt-tRNA modopathies

Disease	Method	Target	Effect	Reference
MELAS	Removal of 3243A > G mtDNA by mitoTALEN	3243A > G iPS cells	Eliminated 3243A > G mtDNA and restored much of respiratory functions	Yang et al. 2018
MELAS	Taurine supplementation (phase III trial)	MELAS patients with 3243A > G or 3271T > C	Prevented stroke-like episodes, but did not stop progression of brain atrophy	Rikimaru et al. 2012; Ohsawa et al. 2019
MELAS	Overexpression of *MTO1*	3243A > G cybrids	Restored τm^5^U modification rate but not tRNA^Leu^ level and partially restored the O_2_ consumption rate	Tomoda et al. 2023
MELAS	Overexpression of C-terminal of LARS2	3243A > G cybrids	Restored much of the O_2_ consumption rate	Perli et al. 2014
MERRF	Removal of 8344 A > G mtDNA by mitoTALEN	8344A > G cybrids	Improved heteroplasmy level to 60%–80% and restored OXPHOS	Hashimoto et al. 2015
MERRF	Overexpression of *MTO1* or *TRMT61B*	8344A > G homoplasmic myoblasts	Restored mitochondrial translation level	Richter et al. 2018
RILF	Knockdown of CLPP protease to slow down degradation of mutated MTU1	HEK293 cells expressing RILF-mutant MTU1	Partially restored the MTU1 protein level and the τm^5^s^2^U modification level	Ahmad et al. 2024

There are approximately 100–10,000 copies of mtDNAs within a cell (Wachsmuth et al. 2016). In patients with mitochondrial disease caused by mtDNA mutations, pathogenic mtDNAs are usually present alongside wild-type mtDNAs (heteroplasmy), although the mutant load varies across cells, tissues, organs, and ages (Greaves et al. 2014; Stewart and Chinnery 2021). When the ratio of mutated DNA to wild-type DNA is low, the wild-type mtDNA compensates for the mutated mtDNA. A threshold effect (in which a threshold, usually >60% mutant mtDNA, is exceeded before symptoms manifest) is a feature typical of heteroplasmic mtDNA diseases (Gorman et al. 2016); therefore, attempts have been made to shift the heteroplasmic ratio below this threshold.

Mitochondrial replacement therapy is a powerful strategy that uses enucleated donor oocytes to replace defective maternal mitochondria: The aim is to prevent inheritance of mtDNA mutations (Wang et al. 2014). Some studies report successful pregnancies in humans, but information is limited (Zhang et al. 2017). These techniques require a combination of genetic material from three individuals, which has raised ethical concerns and legal issues. For example, in the UK, mitochondrial donation is a highly regulated process, whereas in many other countries, it is currently not allowed due to ethical reasons (Russell et al. 2020).

Transmitochondrial cybrids are cells in which mutant mtDNAs derived from mitochondrial disease patients are transferred into cells lacking mtDNA. Cybrids are used to study the effects of mutant mtDNA without any differences in the nuclear DNA background. A direct approach to curing heteroplasmic mtDNA diseases is the use of mitochondria-targeted, sequence-specific DNases to delete the mutant mtDNAs. This method is based on the premise that double-strand breaks in mtDNAs induce rapid degradation instead of their repair; the residual wild-type mtDNA then replicates and restores normal mtDNA levels. CRISPR/Cas is not applicable to mtDNA because the CRISPR/Cas guide RNA has to be imported into mitochondria, and no methods for RNA import are available. In contrast, mitochondria-targeted transcription activator-like effector nuclease (mitoTALEN) was used successfully to reduce the amount of 8344A > G mtDNA in MERRF patient-derived cybrids; this increased the wild-type mtDNA load to 60%–80% and restored oxygen consumption (Table 3; Hashimoto et al. 2015). Moreover, mitoTALEN completely eliminated 3243A > G mtDNA in MELAS patient-derived induced pluripotent stem (iPS) cells, resulting in normal mitochondrial respiration in these cells (Table 3; Yang et al. 2018). Although mitoTALENs are successful at a culture cell level, their in vivo safety and efficacy profiles are yet to be assessed. For in vivo investigations, a mouse model of mtDNA heteroplasmy would be useful; however, due to extreme difficulties associated with manipulation of mtDNA, only a few mouse models of mtDNA heteroplasmy are available, and all are unrelated to mt-tRNA modopathies (Kauppila et al. 2016; Stewart 2021; Tani et al. 2022). The use of mtDNA base editors may facilitate the development of such heteroplasmic mt-tRNA modopathy mouse models; these editors are discussed below.

Although most mtDNA variants are heteroplasmic, some are homoplasmic (Boule et al. 1992). MitoTALENs cannot be applied to such homoplasmic variants due to a lack of wild-type mtDNA; therefore, mtDNA base editors may be useful. An mtDNA base editor is a protein composed of a mitochondria-targeting signal, a TALE or zinc finger array for sequence-specific DNA binding, and a C-to-U (uracil) or A-to-I (inosine) editing domain (Gammage et al. 2018; Cho et al. 2022; Kar et al. 2023). After targeted C-to-U or A-to-I DNA editing, the mtDNA replication process changes the U to T or I to G. One serious concern with mtDNA base editing is off-target effects, which include “bystander” editing (i.e., editing of other bases near to the target site). With respect to the most common mtDNA pathogenic variant, 3243A > G, reversing the G to A by C-to-U editing of the complementary strand remains challenging because multiple bystander events may occur given its sequence context (Kar et al. 2023); however, substantial efforts are being made to reduce off-target effects, including a reduction in expression of the mtDNA base editor (Silva-Pinheiro et al. 2023). The result of this reduction in expression is that the mtDNA base editor succeeded in knocking out each of the 13 mtDNA-encoded protein genes in cultured cells and allowed the development of heteroplasmic *Atp6* mutant mice (Silva-Pinheiro et al. 2023). Thus, the application of such technologies to generate MELAS or MERRF model mice is awaited eagerly; such models could be used to investigate the efficacy and safety of various curative treatments.

Clinical trials of high-dose oral taurine supplementation for MELAS patients have been reported (Rikimaru et al. 2012; Ohsawa et al. 2019). MELAS mutations cause loss of the τm^5^U modification in mt-tRNA^Leu(UUR)^ (Yasukawa et al. 2000b), and the addition of taurine to the culture medium ameliorates reduced oxygen consumption by patient-derived cells (Rikimaru et al. 2012). A phase III clinical trial revealed that 52 weeks of oral taurine supplementation led to a marked reduction in stroke-like episodes, without any adverse effects; however, brain atrophy remains progressive, suggesting that this treatment is not a cure for MELAS (Ohsawa et al. 2019). Nevertheless, due to the reduced frequency of stroke-like episodes, Japan approved taurine supplementation as a treatment for MELAS from 2019.

Although the 3243A > G mutation in mt-tRNA^Leu(UUR)^ makes it a poor substrate for the MTO1–GTPBP3 complex, τm^5^U in MELAS patient-derived cultured cells was restored almost completely by overexpression of *MTO1* (Tomoda et al. 2023); however, *MTO1* overexpression led to only partial recovery of oxygen consumption, likely due to unchanged degradation of the mutant mt-tRNA^Leu(UUR)^ caused by its aberrant tertiary structure.

Overexpression of mt leucyl-tRNA synthetase (LARS2) or its C-terminal domain in MELAS patient-derived cells led to partial restoration of the oxygen consumption rate (Perli et al. 2014). The overexpressed LARS2 C-terminal domain bound stably to mt-tRNA^Leu(UUR)^ but did not rescue the reduced mt-tRNA^Leu(UUR)^ level. Thus, it remains unclear how the overexpressed LARS2 recovers mitochondrial function.

Overexpression of *MTO1* in MERRF patient-derived cells restored mitochondrial translation almost completely (Richter et al. 2018). In MERRF patient-derived cells, in addition to τm^5^s^2^U, m^1^A at tRNA position 58 was also lost from mt-tRNA^Lys^ (Fig. 2D). Interestingly, overexpression of TRMT61B, the methylase for m^1^A (Chujo and Suzuki 2012), restored not only m^1^A levels, but also mitochondrial translation levels, without altering the steady-state level of mt-tRNA^Lys^ (Richter et al. 2018); however, before we can consider the potential application of *TRMT61B* overexpression to other mitochondrial diseases, we must elucidate how overexpression of *TRMT61B* promotes mitochondrial translation.

RILF, a transient and acute type of liver failure that occurs during infancy, is caused by various mutations in mt-tRNA 2-thiouridylase MTU1, which is responsible for 2-thiolation of τm^5^s^2^U modification (Umeda et al. 2005; Zeharia et al. 2009). Importantly, RILF mutations lead to the degradation of MTU1 by the CLPX–CLPP protease complex, although most of the MTU1 mutants can incorporate 2-thiolation when the amount of mutant MTU1 is restored by overexpression. In addition, inhibition of CLPP can (partially) restore the 2-thiolation level (Ahmad et al. 2024). Considering that RILF is both acute and transient, and has a survival threshold at 10% 2-thiolation (Ahmad et al. 2024), mitigation might be possible via transient inhibition of CLPP, or transient administration of an *MTU1* expression vector, to push the 2-thiolation level above the 10% threshold.

## CONCLUSION AND FUTURE CHALLENGES TO CURE mt-tRNA MODOPATHIES

During the last four decades, there has been much progress in the field of mt-tRNA modification, including the identification of mt-tRNA modifications, the modifying enzymes involved, and the pathogenic mechanisms that drive mt-tRNA modopathies. Moreover, based on basic mechanistic insights into pathogenesis, different methods have been developed to restore mitochondrial translation, albeit mostly at the culture cell level. However, challenges remain with respect to the application of such methods. To this end, future studies should use the previously generated animal models of mitochondrial disease (Table 2), or newly develop disease models, to investigate the in vivo safety and efficacy of these methods. Previously, an mtDNA heteroplasmic mouse line with a mutation in *mt-tRNA*^*Ala*^, which is unrelated to mt-tRNA modopathy, was developed (Kauppila et al. 2016). Importantly, this model was used to investigate the ability of a mitoTALEN to degrade the mutant mtDNA, and increase the wild-type DNA ratio, in heart and skeletal muscle (Bacman et al. 2018). Regarding the mtDNA 3243A > G heteroplasmy that causes MELAS, a mitoTALEN was developed to eliminate the mutant mtDNA in human cultured cells (Yang et al. 2018); however, if we are to use mitoTALEN as a therapy for heteroplasmic MELAS, we must first develop a MELAS mouse model by base-editing the mouse *mt-tRNA*^*Leu(UUR)*^ gene to generate a humanized *mt-tRNA*^*Leu(UUR)*^, followed by the introduction of a heteroplasmic mutation equivalent to 3243A > G. Then, the mouse model could be used to rigorously test the in vivo efficacy and safety of mitoTALEN as an agent to remove 3243A > G mutated mtDNA.
